# Dynamic Feature Dataset for Ransomware Detection Using Machine Learning Algorithms

**DOI:** 10.3390/s23031053

**Published:** 2023-01-17

**Authors:** Juan A. Herrera-Silva, Myriam Hernández-Álvarez

**Affiliations:** Departamento de Informática y Ciencias de la Computación, Escuela Politécnica Nacional, Ladrón de Guevara E11-25 y Andalucía, Edificio de Sistemas, Quito 170525, Ecuador

**Keywords:** classification, dataset, dynamic, analysis, encryptor, features, locker, machine learning, ransomware

## Abstract

Ransomware-related cyber-attacks have been on the rise over the last decade, disturbing organizations considerably. Developing new and better ways to detect this type of malware is necessary. This research applies dynamic analysis and machine learning to identify the ever-evolving ransomware signatures using selected dynamic features. Since most of the attributes are shared by diverse ransomware-affected samples, our study can be used for detecting current and even new variants of the threat. This research has the following objectives: (1) Execute experiments with encryptor and locker ransomware combined with goodware to generate JSON files with dynamic parameters using a sandbox. (2) Analyze and select the most relevant and non-redundant dynamic features for identifying encryptor and locker ransomware from goodware. (3) Generate and make public a dynamic features dataset that includes these selected parameters for samples of different artifacts. (4) Apply the dynamic feature dataset to obtain models with machine learning algorithms. Five platforms, 20 ransomware, and 20 goodware artifacts were evaluated. The final feature dataset is composed of 2000 registers of 50 characteristics each. This dataset allows for a machine learning detection with a 10-fold cross-evaluation with an average accuracy superior to 0.99 for gradient boosted regression trees, random forest, and neural networks.

## 1. Introduction

Because of the amount of sensitive information stored on both devices and the cloud while transferring over the network, malware detection, especially ransomware, has become a primary research topic in recent years. A ransomware-like attack uses a set of stages to infect a system; it starts with the device’s distribution and infection. This malware searches for files to infect. It encrypts files, requests ransom, and threatens exposure to the affected victim’s sensitive information in case of non-payment. Ransomware encrypts the files of its victims’ computers for a short time to hijack the information and ask for a ransom. Standard methods of discovering the malware’s signature do not work because the virus has a continuous evolution, making detection of this virus’s action difficult. Due to this threat’s many signatures, traditional signature-based detection techniques do not work well with ransomware.

Individuals and companies can prevent ransomware attacks using rules considered best practices, including using commercial antivirus to protect web applications, disabling macro scripts, removing unauthorized apps, and creating sub-networks to reduce ransomware diffusion. This online source (https://techviral.net/ransomware-encrypted-file-extensions/, accessed on 14 May 2022) presents a complete list of ransomware virus extensions that could be useful for static analysis. The most common delivery system for ransomware is using an email attachment and phishing techniques. Hackers persuade individuals to open a malicious link or an attachment that allows ransomware to enter their computers.

However, it is important to continue researching methods to detect the ever-evolving ransomware threat. The solutions that should be implemented commercially must have the flexibility and the ability to detect patterns from previous attacks to predict new variants using methods such as the ones described in this paper. This type of detection could be the only solution given this threat’s constant change.

Detecting ransomware is more complex than identifying general malware because of the ever-increasing number of ransomware with different signatures. Therefore, new protection mechanisms must focus on dynamic ransomware operations before encrypting files [1].

The authors found that there are no readily available public databases with dynamic feature information on this type of attack. The few existing data are challenging to use because they are not described in enough detail or only consider a few dynamic parameters. In this context, this work proposes to create and publish a feature dataset with all the information necessary for its use. This paper also details the parameters chosen as dynamic features and the criteria applied in the selection. Additionally, the attributes were tested to avoid redundancy due to correlation among the data.

Our research aims to create a feature dataset that associates the ransomware samples used with the most distinctive dynamic virus attributes to detect them before the attack does its damage. This work presents this material to make it available to the scientific community and thus to contribute to advancing the fight against this computer threat. The dataset is used to generate machine learning models that allow for early virus detection and achieve a proactive response that minimizes the harm this malware can cause.

The dataset produced in this study has relevant and low-correlated characteristics associated with ransomware generated during run-time. The parameters involved in creating the dataset are based on cuckoo reports that generate 326 features; from these attributes, 50 were chosen because they have the most pertinent information about the ransomware threat. This selection was made after an analysis of the role of each parameter during an attack. Several datasets were tested using different combinations of the features applying machine learning algorithms. With the final 50 selected characteristics, a ransomware feature dataset was created.

**Figure 1:**
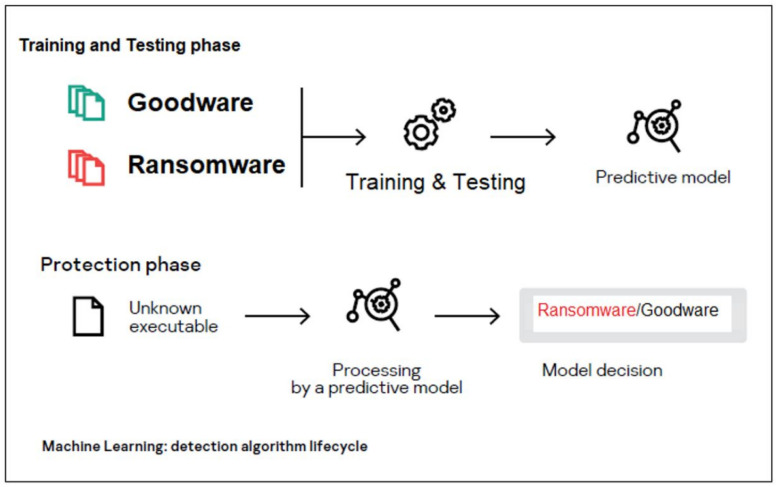
Detection lifecycle.

The virus’s behavior is analyzed through our dataset using machine learning algorithms, as shown in Figure 1. The dataset is used to create machine learning models. They are evaluated with a 10-fold cross-evaluation approach to prove their efficacy. For the protection phase, the models with the best performance were chosen.

The present research has the following objectives:

1. Execute experiments with goodware, encryptor ransomware, and locker ransomware to generate JSON files with dynamic parameters that characterize the artifacts. For this purpose, programs were executed in an isolated environment with tools such as cuckoo sandbox.
2. Analyze and select the most relevant and not redundant dynamic parameters obtained, running the artifacts in an isolated environment for identifying encryptor and locker ransomware from goodware.
3. Generate a dynamic feature dataset that includes the chosen parameters for samples of different artifacts in several Windows platforms.
4. Apply the dataset to the generation of models obtained with machine learning algorithms to detect encryptor and locker ransomware using different combinations of features to determine the selection of parameters that gives the best algorithm performance. These models will detect the ransomware before the information is encrypted and hijacked.
5. Make this dynamic feature dataset publicly available to be used by the community to generate machine learning models to be applied in ransomware detection.
6. Our hypotheses are:
7. It is possible to build a feature dataset obtained from running encryptor and locker ransomware and goodware corresponding to several artifacts in various Windows platforms.
8. The features will deliver enough information to produce machine learning models to detect encryptor and locker ransomware, with performance over the state-of-the-art values. Their deployment will allow for early detection of ransomware to minimize the possible damage.

The present document consists of six sections. The first one is this introduction. Section 2 is about related work, describing current research, the most used features, datasets, and their sources. Section 3 describes the materials and methods used in our work. Section 4 presents the generated feature dataset and the modeling using the selected parameters as input to machine learning algorithms to classify goodware, encryptor, and locker ransomware and their respective results. This section also presents a structure for the deployment of the best models. Section 5 offers a discussion of the contributions of the research. Finally, Section 6 exposes the study’s conclusions.

## 2. Related Work

The work on situational awareness of ransomware attacks [2] identifies parameters for detecting and preventing this attack. This paper presents analysis tools used in different studies, such as mainly cuckoo sandbox [3,4,5,6,7,8,9] to create an isolated environment to safely run the virus, process monitor logs [6] and watchdog module [10].

Similarly, as in [11], an analysis is performed on a set of parameters related to ransomware attacks. The most commonly used metrics are convergence region (ROC) against file encryption, CPU utilization, valid positive rate (TPR), false-positive rate (FPR), accuracy, and recovery. On the other hand, according to the RWGuard system [12], the parameters that can influence the detection of ransomware are required input and output packets, behavior, and CPU processing.

Ransomware detection investigations mention the main features that are used [2]: inspection of content similarity and entropy; checking C&C communications [13]; examination of the file system activity that can include changes in the master file table (MTF) and I/O request packets (IRP) [14]; monitoring registry values; detection of privilege escalation requesting for administrative rights [15]; monitoring API and DLL calls; finding modifications of the master boot record (MBR), monitoring specific file types, file paths, or directories to see an unusual increase in particular extensions, such as .locky [16], and monitoring network activity. The authors in [17] use ransomware opcodes (machine language instructions) for machine learning detection of the virus.

Most authors only use a partial set of these parameters to feed a conventional algorithm or a machine learning classifier. There is not enough explanation of the parameters and identifiers’ descriptions, and most of the time, there is no clarification of why they are considered. Thus, studies that generate dynamic feature datasets provide only an overview of the characteristics used in the ransomware attack detection process; they do not provide a detailed description, and most feature datasets are also unavailable. The drawback is that it is unclear which specific features inside each category are included in the detection; therefore, their experiments are challenging to replicate.

In [18], a method has been introduced to detect ransomware on virtual servers. Volatile memory dumps obtained from forensic memory analyses are analyzed to create meta-characteristics. The experiment was conducted using the volatility foundation and random forest classifier as a machine learning model [19,20,21].

As [22] states, the application of intelligent algorithms to detect ransomware is in an early stage but is growing. New perspectives of future developments are still ahead in this research area. As shown in Table 1, the following studies use machine learning algorithms to classify ransomware from goodware: [4,10,12,13,14,16,17,18,20,21].

Table 1 shows that only one study works in the Android operating system [3]; one is applied over a software defined network (SDN) [23], all the others analyze at least one Windows platform, and one also researches over the Linux server [24,25].

Table 1 shows that only one paper [26] delivers an available dynamic feature dataset. Still, its features are only a partial subset of all the dynamic features. They are related to I/O operations, entropy, and large block addressing to hard disks (LBA).

The performance of the presented studies uses several metrics. It varies from an accuracy of 70% to a maximum of 99.7%; a maximum F-measure of 0.99; detection rate with values from 90% to 98.25%; one paper presents a ROC of 0.995; and another shows a response time of 100 ns to disrupt the connection for C&C communication before the encryption is made.

Only the last two apply artificial neural networks (ANN) such as convolutional neural networks (CNN) and bi-directional long short-term memory (Bi-LSTM); the others implement supervised algorithms such as naive Bayes (NB), support vector machine (SVM), sequential minimal optimization (SMO), logistic regression (LR), decision tree (DT), random forest (RF), simple logistic (SL), decision trees (DT), K-nearest neighbor (KNN), and gradient boosting decision tree (GBDT) [27,28,29,30]. 

The ransomware sample sources configuring the ransomware–goodware datasets used in these studies are mainly VirusShare (https://www.impactcybertrust.org/dataset_view?idDataset=1271, accessed on 18 May 2022), theZoo (https://github.com/ytisf/theZoo, accessed on 22 June, 2022), VirusTotal (https://www.virustotal.com/gui/home/upload, accessed on 18 June, 2022), and hybridanalysis.com (https://www.hybrid-analysis.com/, accessed on 6 April, 2022). They form repositories with different ratios between the number of benign and ransomware artifacts. Some repositories include general malware artifacts. Therefore, the datasets are a collection of malware and goodware obtained from previously mentioned sources. Some authors also use logs collected by users. These datasets are the sample collections used to apply detection algorithms.

As far as the authors know, there is no accessible dataset with a robust set of dynamic features obtained from running the virus in an isolated environment. This lack of a feature dataset makes it challenging to develop detection and prevention solutions for the constantly evolving signature-changing ransomware [31]. When other authors use dynamic features, they only apply some attributes, for example, attributes related to the network, API and DLL calls, or file systems [32,33,34,35,36]. For better classification results that detect new variants not present in the training dataset, it is necessary to use a more complete description of the ransomware activities delineated by the presence of all the relevant dynamic features.

A complete dataset of dynamic features is needed to be used as a basis for intelligent machine learning detection with the capability to produce models to identify this threat before it causes damage. For this reason, this research deals with these two issues: the generation of a relevant feature dataset and its use to produce machine learning models to differentiate ransomware from goodware.

## 3. Materials and Methods

This research conducts a dynamic analysis using a sandbox, specifically cuckoo. In this section, the authors briefly establish a background related to machine learning algorithms and ransomware analysis (static and dynamic). Additionally, this section reports the characteristics of the cuckoo sandbox, the feature extraction tool, the test settings, and the chosen dynamic features that form the input vector for the machine learning algorithms.

### 3.1. Machine Learning Algorithms

In this study, we tested machine learning algorithms to generate the models to recognize ransomware. The algorithms used are shown in Table 2; this research chose the algorithms: Gaussian naive Bayes, random forest, gradient boosted trees, and artificial neural networks.

### 3.2. Ransomware Analysis

In general, malware analysis is the study, observation, and dissection of malicious software to determine its purpose, origin, and functionality [47,48]. The analysis of this type of software is necessary to develop techniques that facilitate the detection of malware and tools that allow it to be counteracted [49]. The analysis could be classified as static or dynamic.

#### 3.2.1. Static Analysis

This analysis focuses on studying a malicious software artifact without running it [47,48]. Within a basic static analysis process, several activities are carried out, such as evaluating the software artifact in question within various antiviruses, searching within a binary file for readable text strings, and examining the artifact’s metadata, among others.

One of the advantages of using this type of analysis is that it allows for an in-depth view of the content and behavior of an artifact. However, some disadvantages can make this type of analysis challenging, such as code obfuscation or if the artifact in question uses self-modifying code techniques [47].

Some of the methods used in this type of analysis are:

Disassembly: It uses tools that allow reverse engineering to be carried out on the device in question [48]. With this technique, the intention is to obtain the instructions of the malware in assembly language from the machine code that contains the malicious software to analyze the instructions and determine the behavior of the artifact [47].

Information extraction: This strategy involves extracting the information embedded in the malicious artifact without necessarily performing reverse engineering. This process includes removing readable text strings within the artifact or searching for information based on the file extension [47].

Use of antivirus: It simply passes the malicious artifact through several antiviruses from different providers [47,49]; most antiviruses use fixed signatures of known threats.

#### 3.2.2. Dynamic Analysis

Dynamic analysis focuses on executing the malicious artifact within a controlled environment. This execution allows the researchers to observe and monitor the behavior of the malware in the controlled testbed and determine the changes it has made on it [47,48,49]. Since a malicious artifact is going to be executed in this analysis, it is necessary to have a safe environment to be able to guarantee that, after running it, counterproductive results are not obtained, such as the infection of neighboring networks or the corruption of the computer that is running the malware. For this purpose, simulators, emulators, or sandboxing are used [49]. In this way, the dynamic analysis seeks to obtain some information on the execution of the artifact in question, such as:

- System calls;
- Processes and process trees;
- Modified system registries;
- Files and directories created, modified, or deleted;
- Network connections established;
- Network protocols used.

Our research focuses on the dynamic analysis of ransomware using a sandbox to obtain information on ransomware behavior and goodware software artifacts to conduct dynamic analysis using a cuckoo tool. In addition, the authors describe a feature extraction program developed for this purpose. During execution, the artifacts yielded 326 dynamic features that describe what the artifact does while running inside an isolated operating system. Some of these features are related to ransomware activities and are pertinent for detecting this malware using machine learning techniques. The researchers analyzed ransomware behavior and chose 50 relevant and not redundant features to feed the learning algorithms to produce an accurate classification.

### 3.3. Cuckoo Sandbox

A sandbox is a quarantined environment that enables the malware to be executed by implementing specific security mechanisms to guarantee the environment’s integrity [50]. A sandbox can store information about the artifact’s behavior run within it. This information is later sent back to the environment where the sandbox analyzes the recorded behavior [51]. The implementation of a sandbox varies depending on what is monitored [50]. A sandbox based on virtual machines is commonly used [51].

A virtual machine can be perceived as a computer embedded within another computer. It has a host operating system that can include one or more guest operating systems so that the guest system cannot directly affect the integrity of the host system. In addition, this type of program creates snapshots that are images of a specific virtual machine at a particular time [48]. With these captures, the state of a virtual machine can be restored once an artifact’s execution and dynamic analysis process has finished [51]. For dynamic malware analysis, it is necessary to have a base snapshot to reverse all the adverse effects that malicious software has caused on a virtual machine. Next, the flow of the analysis of a software artifact with the use of a sandbox is described [51].

9. The host system searches for a free sandbox in case more than one is available.
10. The host uses the base snapshot to reset the selected sandbox to its initial state and starts it.
11. The host establishes a communication channel with the sandbox to monitor and exchange information.
12. The artifact is transferred to the sandbox by the host system and is executed.
13. The host uses multiple tools to monitor and record any activity or change within the sandbox at the network level, file system, memory, registers, and operating system, among others.
14. The host proceeds to save all the information collected from the execution of the artifact in the sandbox into one or multiple files for later review.

For this analysis process to be successful, the sandbox must be as similar as possible to a standard user’s computer. Otherwise, the virus may detect that it is being analyzed and may not run [51,52].

### 3.4. Feature Extraction Tool

**Figure 2:**
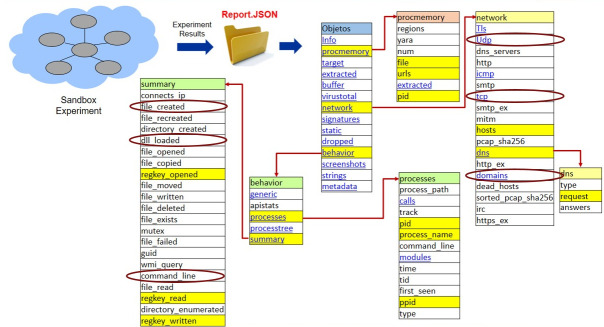
General structure of the JSON report.

Sampling artifacts (goodware and ransomware) and running tests on the cuckoo sandbox system allowed for the creation of a folder containing reports of the different analyses. Figure 2 shows the general structure of the JSON reports generated in the cuckoo sandbox [52].

A report has a tree-based structure. An application to select features in the different levels was developed. For example, the attributes marked with yellow were chosen in the first stage, as seen in Figure 2. The first level contains several categories, such as “Info” or “procmemory”. To begin the extraction process, the application visualizes the type of data stored in each category. The JSON cuckoo sandbox reports are recursively loaded since there were nested directories, and the program looks up every JSON file contained in a given directory.

For instance, the “network” category contains features such as “hosts” and “dns”; “dns” includes the “request” feature. The program extracts all data collected in these features and writes the data contained in a list of any primitive data type or a list of dictionaries to a CSV extension file. This file is the feature vector input in the machine learning algorithms used to generate models to detect locker ransomware, encryptor ransomware, or goodware [52].

### 3.5. Test Settings

**Figure 3:**
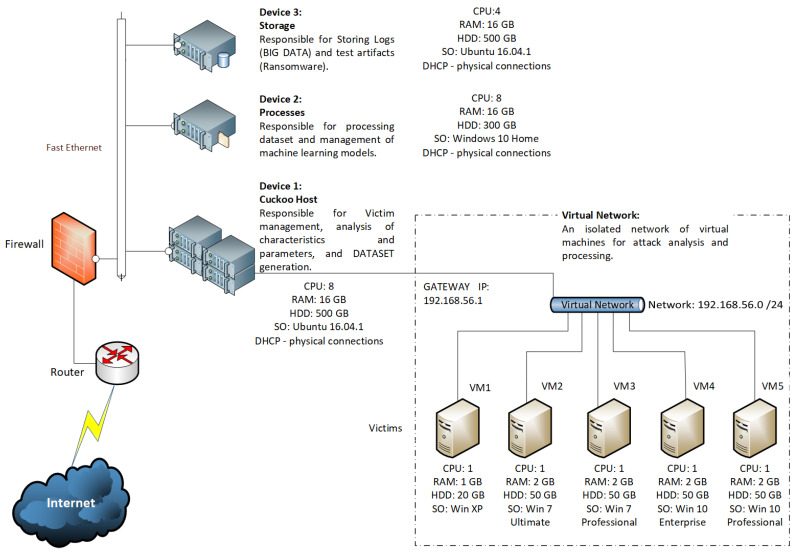
Test environment network topology.

A test scenario was considered in an isolated environment, as shown in Figure 3, to obtain the essential information. Then, our feature extraction tool filtered the attributes required for the dataset conformation. The deployment was based on a safe environment using the cuckoo sandbox tool [53].

Figure 3 shows the final test environment network topology. In this configuration, the experiments use three machines; the first hosts cuckoo, the second CPU processes the models with machine learning, and the third machine is responsible for storing logs (big data) and artifacts for testing. Cuckoo communicates with an isolated virtual network for ransomware processing and analysis composed of CPUs in five platforms: Windows XP Service Pack3, Windows 7 Ultimate, Windows 7 Professional, Windows 10 Enterprise, and Windows 10 Professional.

### 3.6. Selected Features

To generate the dataset, 2000 experiments were performed with 20 ransomware samples and 20 goodware samples. Characteristics were selected if they were affected during the infection process. These characteristics are reflected in Table 3 with the reasons why they are of interest for ransomware detection. In the dataset, the identifiers are assigned depending on the number of times that features have been counted, that is, integer values starting with 0 when there are no records and from 1 onward when there are records.

**Figure 4:**
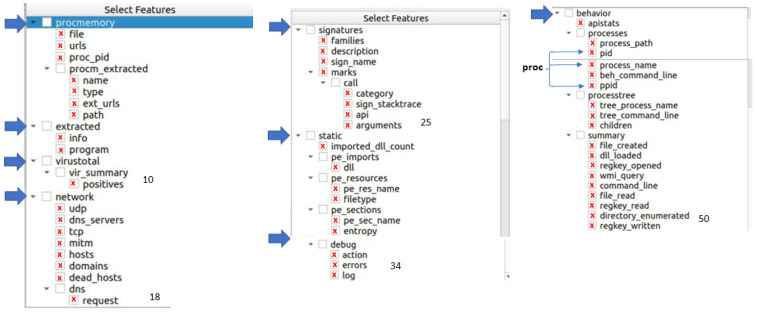
Feature extraction tool GUI for final 50 characteristics.

Figure 4 presents the GUI of the extraction tool used to generate the input vector for the machine learning algorithms, with the final 50 features.

The artifacts (ransomware and goodware) used in the experiments were: 7Zip, Task Manager (taskmgr), API Windows Security Cryptography (cipher), API Windows System Information Registry (regedit), API Windows Volume Management (diskpart), Bitlocker, BitPaymer, Cerber, cmd, Cryptolocker, Cryptowall, Crysis, dllhost, Eris, Windows Remote Desk, GandCrab, gpg, IPScan, Locky, Maze, Microsoft SQL Server Compact, Nmap, Petrwrap, Petya, Phobos, Radamant, RansomX, Ryuk, Satana, services, Sodinokibi, STOP, svchost, Team Viewer, Teslacrypt, Virtual Network Computing VNC, WannaCry, WhatsAppWeb, Winrar, and Wireshark.

## 4. Dataset, Modeling, and Deployment

The dataset and its final features were applied to machine learning algorithms to detect locker ransomware and encryptor ransomware to differentiate them from goodware. The combination of characteristics used was the one that used the criteria related to the effects of ransomware presented in Table 3 with features that have low correlation pairwise.

The final combination of 50 selected characteristics yielded the best algorithm performances. This section will describe the results using two versions of the ransomware features dataset (Step 1 and Step 2), the evaluation of the machine learning models generated, and the deployment using the best models.

### 4.1. Dataset Obtained in Step 1

**Figure 5:**
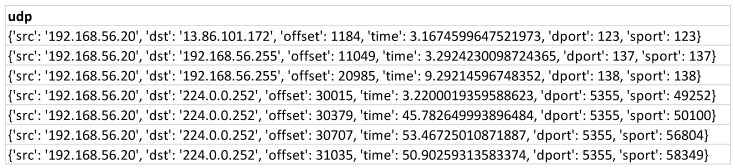
Dataset rows corresponding to a “UDP” feature of an artifact.

In the first step, the information of the dataset is taken from the JSON files generated in the sandbox. Our extraction tool can extract any number of features from each JSON generated by an artifact. For instance, if the authors need to obtain information for one specific characteristic such as “UDP” that corresponds to the connections established through UDP during dynamic analysis; this feature is contained within an object called “network”. It can be observed that this feature does not have one register but multiple rows of information. It is a list of objects. The extraction tool accedes to this list’s content and saves each record in a row within the dataset, as shown in Figure 5.

The same process is applied to extract the rest of the features from the artifact’s JSON file, which is saved in a CSV file. This process is carried out in this stage for different combinations of characteristics. Each corresponding dataset is evaluated with machine learning algorithms to obtain the optimal number of combination of attributes to generate high-performance models.

This research generated datasets with several combinations of characteristics to choose the best option. All the considered features were selected after analyzing whether they relate to ransomware consequences. Selecting relevant attributes from the total of 326 obtained in the cuckoo, the best results were attained for 50 features linked to ransomware’s effects. Table 3 presents a description of these chosen dynamic features. Another criterion to be taken into account is that the attributes have a low correlation ratio pairwise to avoid redundant information. Therefore, these 50 features were chosen, shown in Figure 4 and Table 3, for the dataset obtained in step 1.

### 4.2. Modeling Results with the Dataset Obtained in Step 1

The authors have chosen 50 attributes using the mentioned selection criteria. These characteristics were extracted from 40 artifacts applying ten executions for each artifact in five victim’s devices, giving a total of 2000 JSON files. Because each JSON file has several rows, this first dataset generated in step 1 has 1′424.344 registers after a cleaning procedure to eliminate redundant rows. 

**Figure 6:**
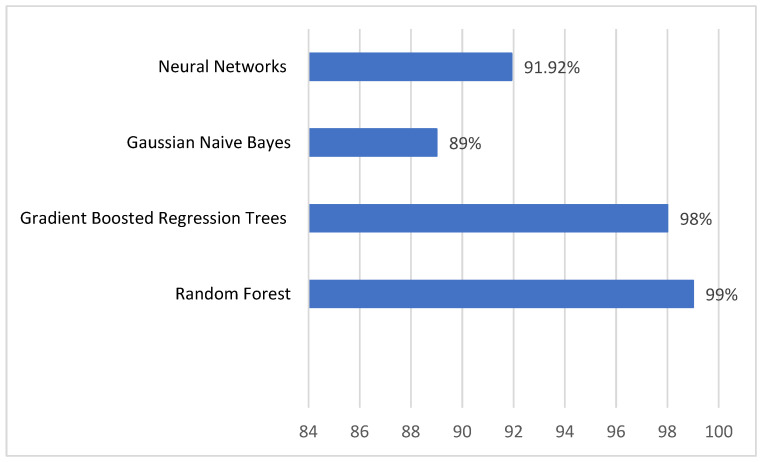
Ten-fold cross-validation accuracy results obtained in step 1.

**Figure 7:**
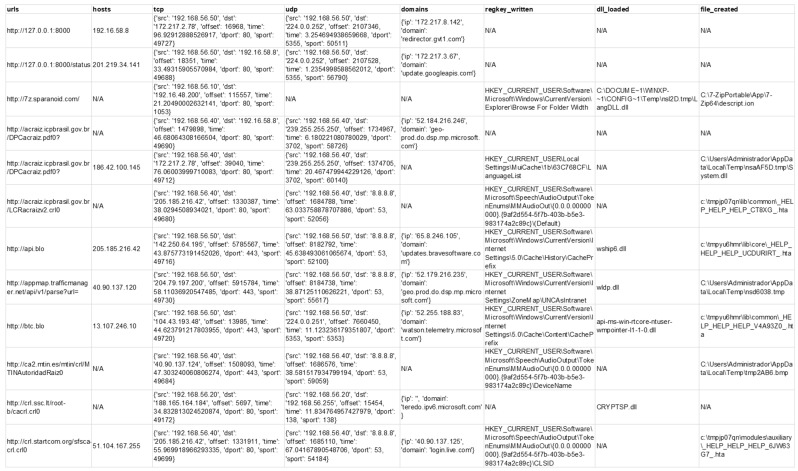
Information of some features for a single artifact—Dataset obtained in step 1.

In the experiments, the researchers established that the algorithms that produce the best performances are random forest, artificial neural networks, and gradient boosted regression trees. In addition, Gaussian naive Bayes was included in these reports because although the yields are lower with this algorithm, the processing time is shorter. The modeling results for this dataset are presented in Table 4 and Figure 6, which contain the logs for the generation of the models for dataset 1 obtained in step 1, using supervised algorithms. Figure 7 corresponds to some features for a single artifact from the dataset obtained in step 1; it shows an example of the information extracted.

The best configuration results for neural networks were found for three layers with 200 neurons, sigmoid activation, and softmax output function. Neural networks are configured to recognize only between ransomware and goodware; therefore, they only present one value for precision, recall, and F1. These results are also shown in Table 4. For random forest and gradient boosted regression trees, the best results, without overfitting, are obtained for 100 estimators, i.e., trees in the forest. G, E, and L mean goodware, encryptor, and locker. Gradient boosted regression trees is the algorithm with the best performance. Still, its processing takes around four hours, making it challenging to update new data for modeling.

The metrics used to evaluate the performance of the machine learning algorithms are accuracy (1), precision (2), recall (3), and F1 (4).

Accuracy is the number of correctly classified data divided by the total number of data samples:

(1) $$\mathrm{Accuracy}=\frac{TP+TN}{TP+TN+FN+FP}$$

where *TP* = true positive; the number of instances where the ransomware was correctly identified. *TN* = true negative; the number of instances where goodware (negative for ransomware) was correctly identified. *FP* = false positive; the number of instances where goodware was classified as ransomware. *FN* = false negative; the number of cases where the model failed to classify the ransomware.

Precision is the positive predictive value, i.e., how many of the positive predictions are correct:

(2) $$\mathrm{Precision}=\frac{TP}{TP+FP}$$

Recall is the true positive rate, i.e., a measure of how many of the positive cases the classifier correctly predicted over all the real positive cases in the data:

(3) $$\mathrm{Recall}=\frac{TP}{TP+FN}$$

F1 is the harmonic mean of precision and recall; it helps to balance the two metrics:

(4) $$F1=2*\;\frac{Precision*Recall\;}{Precision+Recall}$$

The results with the dataset shown in the previous section are satisfactory. However, the file size of this dataset produces longer processing times. Hence, it is neither portable nor efficient to be implemented in the deployment stage. For this reason, the previous dataset was processed to obtain a summary of one row for each JSON file corresponding to an artifact. The study starts with extracting the previously described JSON content to construct this new dataset for the generation of machine learning models.

A more concise matrix is created where the columns correspond to each characteristic extracted from the analyzed artifact. This way, all the information collected throughout the analysis is grouped, and the number of records by columns is counted. A cell with the value “N/A” is not counted. If it has a value other than “N/A”, it is calculated. For example, in Figure 7, all the rows produce a unique row corresponding to one execution. Figure 7 only shows 7 of the 50 selected features. The number of registers for each column in the considered categories is found in each cell.

The researchers proceed to do this with the two thousand experiments with available reports. Using this procedure, a matrix was obtained where each row had information about an artifact. Each row cell corresponds to a feature of that artifact. This process produces a matrix of 2000 rows and 50 columns. This final dataset is included in Supplementary Materials.

### 4.3. Modeling Results with the Dataset, Step 2

**Figure 8:**
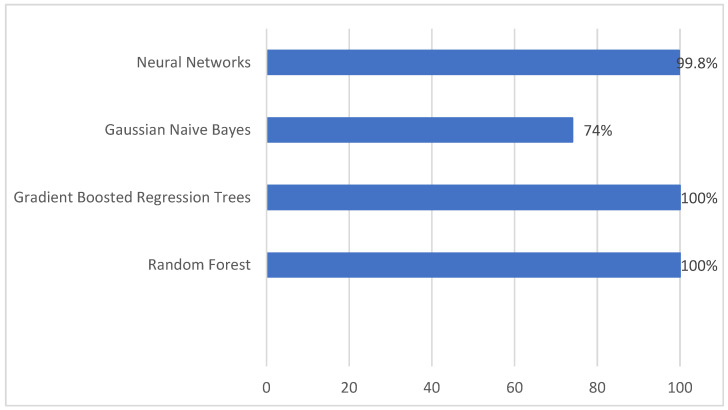
Ten-fold cross-validation accuracy using the dataset obtained in step 2.

For the machine learning algorithms, this study used the parameters specified in the previous section, which are the ones that produce the best performances. For random forest and gradient boosted trees, the performances for 100 estimators or trees is shown. For neural networks, all the models have high performances. The authors chose one similar to the parameters used for neural networks for the dataset obtained in step 1, i.e., with three layers, 100 neurons in each. However, the authors selected SELU as an activation function because, in this case, it runs faster. Table 5 and Figure 8 present the performance results.

The best results are obtained with this second dataset except for Gaussian naive Bayes, which has lower accuracy. Processing times for the model obtention are significantly lower than with the previous dataset. Again, the best performance algorithms are random forest and gradient boosted regression trees, and slightly lesser values were obtained using neural networks with three layers with 100 neurons each. Bayes reduces performance values from 89 obtained in step 1 to 74 obtained with the summarized dataset for 10-fold cross-validation accuracy.

Table 6 summarizes the steps and the CSV dataset’s generated registers.

### 4.4. Deployment

The prediction of new artifacts requires generating a CSV file with the previously described tool for extracting features of a new artifact. Any generated model can be used to make predictions. The content of these files is concise enough to change the directories of CSV files and models to execute the deployment.

**Figure 9:**
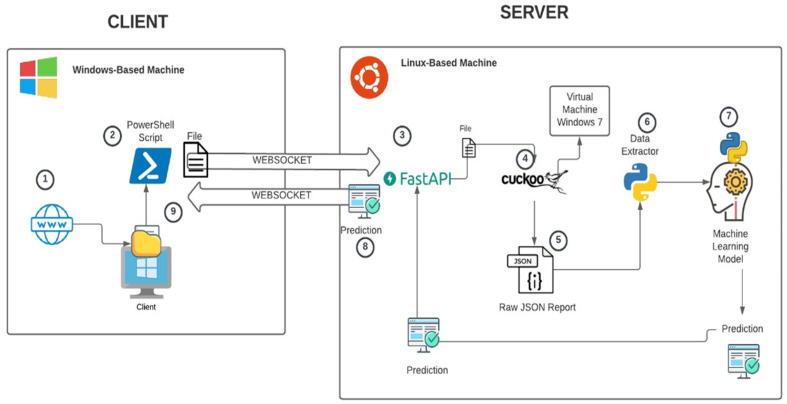
Deployment architecture.

Our architecture allows for analyzing the behavior of an artifact since it is created in a file system. It considers the sandbox environment for the dynamic analysis of an artifact, the information extraction tool obtained from the analysis, and the machine learning models to be used to classify the analyzed artifact, as shown in Figure 9.

The process of analyzing an artifact by deploying the models is detailed below:

1. A file is introduced into the computer, for example, through a network.
2. Using a Powershell script, the introduction (creation) of the file to the file system of the operating system is detected.
3. The client uses the Powershell script to open a WebSocket-type connection with the server and to send the file in question.
4. Once it has received the entire file, the server starts the dynamic analysis process using the cuckoo sandbox tool.
5. After completing the dynamic analysis process, cuckoo sandbox collects all the information. It saves it in a file in JSON format.
6. Once the creation of this file is detected, a variation of the information extraction tool is used to extract the relevant information that will serve as input for the machine learning models.
7. Once the information has been extracted, the feature vector is built and sent to one of the previously trained machine learning models to obtain the classification (prediction) of the analyzed file.
8. The classification (prediction) provided by the model is sent through the WebSocket connection to the client to take action, depending on whether it is ransomware or not.

## 5. Discussion

### 5.1. Contributions of This Work

From Table 1 and Table 2, comparing the characteristics of other research with the present work, it can be inferred that our experiment has several advantages:

— Unlike all the other studies analyzed in the Related Work section, which only use around three types of features, our research uses the full range of related attributes to study artifacts. This full use of different characteristics allows for the recognition of behavior patterns common to ransomware. Therefore, even new variants not initially present in the training set can be detected.

The attributes used by this research are:

- PROCMEMORY: memory management information;
- EXTRACTED: information on executed scripts;
- NETWORK: network data;
- SIGNATURES: predefined patterns that might represent malicious behavior;
- STATIC: static analysis data, including entropy level obtained by the cuckoo sandbox software;
- BEHAVIOR: libraries to which the artifact makes calls, suspicious processes, and affected registry keys;
- DEBUG: actions, errors, and log information recorded during the dynamic analysis.

Table 1 shows that most researchers use only a fraction of all possible types of features available in dynamic analysis, for example, attributes related to the network or API calls that are part of the behavior parameters. For better classification results, it is necessary to use a more complete description of the ransomware activities delineated by the presence of all the related types of dynamic features. For this reason, we chose 50 attributes related to the before-mentioned group of dynamic parameters related to ransomware effects.

— The 10-fold cross-validation accuracy, precision, recall, and F1 values obtained with the final dataset, using random forest and gradient boosted regression trees, are practically perfect, ensuring the threat’s detection with a processing time in the range of seconds. Other studies have detection results comparable to or lower than the ones obtained in our research.
— The dataset that our study delivers is a feature dataset; that is, it is information that is already ready to be used as input to a machine learning classifier to obtain models that can be tested on new data to be categorized. Most studies only mention the ransomware sample sources, e.g., VirusTotal, and the number of ransomware and goodware used in their datasets; they do not deliver their samples dataset nor the features dataset generated with their work. Unlike other studies, we present in the paper the information we produced in a GitHub repository for community use (https://github.com/Juan-Herrera-Silva/Paper-SENSORS, accessed on 2 December 2022).
— The fact that we apply machine learning gives flexibility to our research because this technique allows for the discovery of hidden patterns in the ransomware behavior. Because this study uses the full range of relevant dynamic features without redundant information (with low correlation pairwise), it generates models that recognize patterns corresponding to the locker and crypto-ransomware variants not present in the training set.
— The time it takes for our classifiers to process the samples is in the order of seconds, making it possible to detect the threat and stop it before any damage is achieved.
— The range of platforms used for our study is more complete than the ones used in other studies. The sandbox implementation is executed in Windows XP, Windows 7 Ultimate, Windows 7 Professional, Windows 10 Enterprise, and Windows 10 Professional.

### 5.2. Comparison with Previous Research

The experiments carried out by other authors cannot be reproduced because we do not have enough description of the environment, the datasets, or the specific dynamic parameters with which they work. Other papers only state the number of ransomware and goodware samples used, their sources, such as VirusTotal or VirusShare, and a not enough detailed description of the dynamic parameters applied. Therefore, the information in Table 1 helps compare the methods and results of other studies with the ones in our research. Our results are comparable to or better than those reported in other studies with an almost perfect 10-fold cross-validation accuracy using random forest and gradient boosted trees.

It is important to state that the authors of the present paper initially conducted experiments with partial sets of relevant features in the initial stages of the work. For instance, the researchers used a partial set of relevant features over the training dataset. They obtained results similar to the ones obtained with the complete set of 50 attributes, as seen in Table 7. The characteristics used correspond to procmemory: file_created; behavior (processes and apistarts): regkey_read, dll_loaded; and network: udp, command_line, domain, tcp. With these parameters, the accuracy results for training are good and go from 63.39% to 99.68%. However, using this partial set of parameters, these algorithms have a significantly lower performance in testing with variants not present in the training set, with a higher accuracy at a value of 54% for gradient boosted trees algorithms.

Therefore, the conclusion is that it is necessary to use the 50 chosen attributes that the researchers include in the feature dataset to ensure excellent performance in detecting ransomware variants not present in the training set. This is an essential differentiation of our work, the ability to distinguish new variants due to the combination of the generation of an input vector composed of a complete set of relevant features and the use of machine learning algorithms fed with these attributes.

## 6. Conclusions

The fact that ransomware attacks continue to produce millions in losses worldwide shows that there is much room for improvement in ransomware detection. The present work contributes to some of the still open issues. One of these issues is the necessity of a dataset containing features corresponding to all the ransomware attack patterns that could be used to train supervised algorithms and neural network models. This feature dataset should include all the relevant attributes related to the threat’s behavior and should be open for the development of new machine learning ransomware detection solutions. Our work aims in that direction.

In this article, the authors have developed a dataset composed of the dynamic features of locker and encryptor ransomware and characteristics extracted from goodware. The features were selected with the criteria that they are related to the effects of ransomware. In the literature, it was found that a ransomware dataset with these characteristics was needed because the ones that are publicly accessible do not have dynamic features of the artifacts but only fixed signatures, or their results are challenging to replicate or use for lack of enough descriptive information.

Dynamic analysis is essential for ransomware detection because the run-time attributes have enough information for machine learning early detection of these threats. In our study, since most of these features are shared by diverse ransomware samples, our dynamic analysis can be used even for detecting new variants. For dynamic analysis, the experimentation must be conducted in an isolated environment to protect the network from using a sandbox for artifact execution. For this purpose, cuckoo sandbox was used to create JSON files with nested information of the dynamic features. The features were selected using criteria related to the role of each attribute in the ransomware attacks and the results of experimentation with machine learning algorithms aiming to obtain the best performances. The JSON file’s total number of features was 326, and the chosen characteristics were 50.

On the other hand, when other authors use dynamic features, they only use some attributes, for example, attributes related to the network, API and DLL calls, or file systems. For better classification results that even detect variants not included in the training set, it is necessary to use a more complete description of the ransomware activities delineated by the presence of all the relevant dynamic features.

In developing the final feature dataset, this research has gone through two steps to categorize three classes: locker ransomware, encryptor ransomware, and goodware. This study created two datasets in two phases. Using our dynamic feature extraction tool, the features were tested, and 50 characteristics were selected because they comply with criteria related to ransomware attacks. They were also tested to have a low pairwise correlation to avoid redundant information. In the trials, the study found that high performances for the machine learning algorithms were obtained for these 50 characteristics and the machine learning algorithms mentioned in Section 4. The researchers used 20 ransomware artifacts and 20 goodware families tested with ten experiments, each over five platforms, to produce a dataset with 1′424.344 rows. For this dataset, there were several rows corresponding to one JSON. The best performance results were obtained with gradient boosted regression trees with values of 0.98 for 10-fold cross-evaluation accuracy. However, processing times for machine learning model generation were high because it took in the range of 4 h to obtain the models.

To generate a more portable, efficient, and concise dataset without losing relevant information, the research developed a process for synthesizing all the rows corresponding to one JSON into one row. This way, using the information provided for the previous repository, the study obtained a second dataset with 2000 records corresponding to forty families and ten experiments for each artifact over five platforms. Using this dataset, performance results for our models improved even more for gradient boosted regression trees, random forest, and neural networks because they reached values close to perfect detection for ransomware. The reported accuracy presented in the literature for ransomware detection gives 0.997 as a maximum value; thus, our models have comparable or better performance. Additionally, processing times were reduced from hours (using the first dataset) to seconds using the summary dataset obtained in step 2.

In the deployment, predicting new artifacts requires applying the generated models, whether in the repository or not. The programs allow for changing the directories of CSV JSON files and models to readily execute them in the production stage.

This dataset is available for public access along with the present article and in the GitHub repository (https://github.com/Juan-Herrera-Silva/Paper-SENSORS, accessed on 2 December 2022). This information can be a starting point for generating new methods of detecting ransomware. As the final feature dataset is public access, the authors hope that the scientific community can use, improve, modify, and share this knowledge.

## Figures and Tables

**Table 1:** Characteristics of dynamic analysis solutions.

Study	Features Used in Dynamic Analysis	Machine Learning Based/Algorithms Used	Dataset is Composed of Samples of	Feature Dataset Made Available	Platforms	Performance
[3]	Filesystem and registry in Windows. Permission monitoring in Android.	No	Ransomware of 25 families	No	Windows 10/Android	Not mentioned
[4]	API calls, Registry Key Operations, File/Directory System.	Yes/NB, and SVM	582 ransomware of 11 families, and 942 goodware	No	Windows	ROC: 0.995
[5]	File system, Access Patterns, and I/O Data Buffer Entropy.	No	148,223 general malware	No	Windows	Detection rate 96.3%
[6]	File System, I/O monitoring	No	715 ransomware	No	Windows 7	Detection rate 96.7%
[7]	HTTP traffic characteristics	No	750 CryptoWall 4.0 ransomware traffic–750 Locky ransomware traffic	No	Windows	Detection rate 97–98%
[9]	API Calls	Yes/SVM	588 logs, 312 goodware and 276 ransomware logs	No	Windows	Accuracy 97.48%
[10]	Entropy analysis	No	Not mentioned	No	Windows	Accuracy 92%
[12]	IRP	Yes/NB, LR, DT, RF	261 benign and malicious processes	No	Windows	Accuracy: NB: 80.07%, LR: 81.22%, DT: 89.27%, RF: 96.55%
[13]	API Calls	Yes/RF, SVM, SL, and NB	168 ransomware	No	Windows 7	Maximum accuracy SL: 98.2%
[14]	Command and control (C&C) server	Yes/RF	265 ransomware related flows	No	Windows	Accuracy with 10 fold cross validation 87%
[15]	Portable Executable (PE) File	No	450 ransomware	No	Windows	Accuracy 70%
[16]	Network Traffic	Yes/DT (J48 classifier)	210 ransomware, 264 benign	Dataset sample showed	Windows	Maximum F-measure 96.8%
[17]	Ransomware Opcodes (Machine Language Instructions)	Yes/DT, RF, KNN, NB, GBDT	1787 ransomware	No	Windows	Maximum accuracy 99.3%
[18]	API Calls	Yes/SVM, DT, RF, GBDT	360 ransomware, 532 general malware, and 460 benign software	No	Windows	Maximum Accuracy 96.1%
[19]	API function calls, counts of the behavioral features, and counts of the memory features	No	1000 ransomware, 1000 benign software	No	Windows XP	Detection rate 90%
[20]	API Calls, File/Directory System, Shannon’s Entropy of File Writes	Yes/LR, SVM, RF, GBDT, ANN	574 ransomware	No	Windows 7, Windows 8.1	Detection rate 98.25%
[21]	Selects key features using Multi-Objective Grey Wolf Optimization (MOGWO) and Binary Cuckoo Search (BCS) algorithms	Yes/NB, RF, and SMO	582 ransomware, and 942 goodware	No	Windows	Accuracy NB: 79.3% RF: 82.67, SMO: 82%
[23]	C&C communications	No	Database of malicious URLs	No	-	Time to disrupt the connection: 100 ms
[24]	Master File Table (MTF) and I/O Request Packets (IRP)	No	Logs with 2000 user activity and 2000 ransomware activity	No	Not mentioned	Accuracy 97.4%
[25]	I/O operation, LBA, and Entropy	Yes/RF, SVM, KNN, CNN	7 ransomware families	Yes	Windows 7, Windows Server 2008	F-measure from 0.57 to 0.99
[26]	Semantic Information from Logs	Yes/Bi-LSTM	Logs	No	Linux Server, Windows 7	Accuracy 96.5–99.7%

**Table 2:** Machine learning algorithms.

Algorithm	References	Characteristics
Random Forest	[37,38,39,40,41]	This algorithm is an ensembled method combining tree predictors so that each tree depends on the values of an independently sampled random vector and has the same distribution for all trees in the forest. It can improve performance compared to independent decision trees. The random forest algorithm uses a collection of decision trees to vote and predict the input data class.
Gradient Boosted Trees	[42,43,44]	This algorithm is based on an ensemble of decision trees to improve the performance of each separate tree, considered individually as weak learners. The algorithm applies gradient augmentation algorithms and generates trees sequentially in a way that complements the errors of the previous tree, and this model is not random. Instead, it uses powerful pre-pruning. The trees combined their output results in better models. In the case of regression, the final result is generated from the average of all weak learners.
Naive Bayes	[38,45]	This algorithm generates probabilistic models on target variables. It assumes that input features are independent without pairwise correlation, which is not entirely accurate in most cases. This assumption of uncorrelated attributes makes this algorithm “naive”. The name Bayes comes from the famous probabilistic theorem on which this algorithm bases the generation of the probabilistic model.
Neural Networks	[46]	Neural networks work similarly to a biological brain to recognize patterns of large amounts of data. Multi-layer neural network algorithms received raw data and performed internal processes to extract and select features. For this reason, they had an embedded feature extraction and selection process. A simple neural network includes an input layer, an output layer with the classified variables, and a hidden layer. The layers are connected and form a network of neurons.

**Table 3:** Selected features inside JSON objects.

Object	Description	Feature	Explanation	Reason for Choosing the Feature
PROCMEMORY	It allows for the creation of memory dumps for each analyzed process (before they finish or before the analysis ends).	file	The file was created as a memory dump.	The feature is chosen because this information allows memory forensics monitoring file modifications to find an unusual increase in particular extensions.
		URLs	URLs generated during the execution of memory processes.	The feature is chosen because it stores a list of URLs that can be modeled as suspicious.
		PID	Process identifier.	The feature is chosen because it identifies the generated file (file).
		name	Name of the process in memory.	The feature is chosen because it identifies the name of a possible suspicious process.
		types	Artifact type.	The feature is chosen because it identifies the type of artifact.
		URLs	URLs used by the process in memory.	The feature is chosen because it identifies URLs used in memory by the process.
		path	Memory process storage directory.	The feature is chosen because it identifies the directory.
EXTRACTED	It contains information about scripts executed by an artifact during artifact analysis.	info	Information on the script in question.	The feature is chosen because it identifies information about scripts that could be used during attacks.
		program	Type of program executing the script.	The feature is chosen because it identifies the program that executes possible malicious scripts.
NETWORK	Includes information on the network infrastructure used during the analyses. The data could monitor continuous or unusual communications inside the network.	dns_servers	DNS servers are involved in the analysis.	The feature is chosen due to communication with external domain servers. DNS sub-characteristics (request).
		mitm	Network analysis to verify the type of attacks man-in-the-middle.	The feature is chosen because it identifies attacks man-in-the-middle where a perpetrator is positioned in an exchange between a user and an application.
		dead_hosts	Hosts down during data transmission.	The feature is chosen because it identifies hosts down, which could be one of the effects of ransomware.
		udp	Network analysis of the UDP protocol.	The feature is chosen due to the use of communication via UDP protocol. It corresponds to the udp port number that ransomware could open.
		tcp	Network analysis of the TCP protocol.	The feature is chosen due to the use of communication via TCP protocol. It corresponds to the tcp port number that ransomware could open.
		hosts	Hosts involved in the analysis. Help create blacklists.	The feature is chosen to detect communication with a possible malicious host.
		domain	Domains involved in communication	The feature is chosen because communication with other domains may be a clue for identifying ransomware.
		request	Domains to which requests were sent (queries) DNS.	The feature is chosen because it serves to monitor possible suspicious requests.
SIGNATURES	It contains information about tasks or processes before, during, and after the analysis and the API calls executed by the analyzed artifact.	families	A list of malware family names.	The feature is chosen because it identifies requests that were sent.
		description	Signature description.	The feature is chosen because it supplements information about possible ransomware.
		name	Signature name.	The feature is chosen because it supplements information about possible ransomware.
		category	API call category.	The feature is chosen because it supplements information about possible ransomware. The category of the API calls can be used to model the application behavior.
		stacktrace	Execution stack related to an API call.	The feature is chosen because it supplements information about possible ransomware. The stacktrace of the API calls can be used to model the application behavior.
		api	API call in question.	The feature is chosen because it supplements information about possible ransomware. Some characteristics of the API calls can be used to model the application behavior.
		arguments	Arguments of the API call in question.	The feature is chosen because it supplements information about possible ransomware. Arguments of the API call can be used to model the application behavior.
STATIC	Contains information about a static analysis performed by the cuckoo in case the analyzed artifact is of the type portable executable (PE) that could propagate malicious code.	imported_dll_count	The number of system DLLs imported by artifact.	The feature is chosen because it contains artifact information when the artifact is portable executable.
		dll	System DLL libraries used by the artifact during analysis.	The feature is chosen because it contains artifact information when the artifact is portable executable.
		name	Artifact name.	The feature is chosen because it contains artifact information when the artifact is portable executable.
		filetype	Artifact type.	The feature is chosen because it contains artifact information when the artifact is portable executable.
		entropy	Entropy level of the artifact in question.	Encryption changes the content. Therefore, it has a higher entropy value. This characteristic could help to detect encryption and ransomware; thus, it was selected.
		name	Sections found within the artifact.	The feature is chosen because it contains artifact information when the artifact is portable executable.
BEHAVIOR	It allows for seeing the behavior of the artifact, that is, to see libraries to which it makes calls, suspicious processes, and affected registry keys.	processes	Processes carried out by the device.	The feature is chosen because processes modify the infected system. The authors selected sub-characteristics processes (process_path, pid, process_name, command_line, and ppid).
		processtree	Executed child processes derived from the process tree.	The feature is chosen because processtree contains subprocesses that modify the infected system. The authors selected sub-characteristics processtree (process_name, command_line, and children).
		summary	Summary of files, log keys, directories, and commands involved during the execution of processes.	The feature is chosen because it contains parameters that affect infected systems. The sub-characteristics summary (regKeys) is chosen because register values are modified during a ransomware attack. In addition, the sub-characteristics (file_created, dll_loaded, wmi_query, command_line, file_read, and directory_enumerated) are chosen because ransomware uses these function calls to execute malicious operations in the OS_file system.
DEBUG	It contains information about the analysis performed on an artifact.	action	Actions recorded during the analysis.	This feature is selected because it gives information about the cuckoo and its actions during the experiments’ execution.
		errors	Errors logged during analysis.	This feature is selected because it gives information about the cuckoo and errors during the experiments’ execution.
		log	Various information about the analysis executed.	This feature is selected because it gives information about all the occurrences inside the cuckoo sandbox during the experiments’ execution.

**Table 4:** Performance results for the dataset obtained in step 1.

Algorithm	Average Ten-Fold Cross-Validation Accuracy	Precision (%)			Recall (%)			F1 (%)			Processing Time (Segs.)
		G	E	L	G	E	L	G	E	L	
Random Forest	99.0	87.40	99.40	96.98	91.11	99.28	93.43	89.25	99.34	85.15	5193.67
Gradient Boosted Regression Trees	98.00	83.00	98.85	98.98	85.19	99.07	90.37	84.08	98.96	94.48	14,755.79
Gaussian Naive Bayes	89.00	46.08	92.98	16.47	40.38	96.16	07.19	43.04	94.54	10.00	76.50
Neural Networks	91.92	92.31				90.55		92.12			2804.61

**Table 5:** Performance of the classifiers using the dataset obtained in step 2.

Algorithm	Average Ten-Fold Cross-Validation Accuracy	Precision (%)			Recall (%)			F1 (%)			Processing Time (Segs.)
		G	E	L	G	E	L	G	E	L	
Random Forest	100	99.86	100	100	100	99.831	100	99.93	99.91	100	3.9
Gradient Boosted Regression Trees	100	99.74	100	100	100	99.66	100	99.86	99.98	100	25.47
Gaussian Naive Bayes	74.00	71.11	88.86	52.43	93.62	58.03	38.29	80.83	70.21	4.26	0.15
Neural Networks	99.8	99.8			99.8			99.8			6.99

**Table 6:** Dataset obtained in each step and the number of registers.

Step	Number of Registers of the CSV Dataset File
1	1.424.344
2	2.000

**Table 7:** Performance of the classifiers using a partial set of relevant features over the training dataset.

Model	Accuracy	Precision	Recall	Classification Error
Naive Bayes	63.39%	68.15%	57.86%	16.61%
Neural Networks	98.06%	98.78%	94.71%	1.94%
Random Forest	92.88%	96.39%	65.28%	7.12%
Gradient Boosted Trees	99.68%	99.81%	98.11%	0.32%

(Features: regkey_read, udp, file_created), dll_loaded, command_line, domain, tcp)

## Data Availability

This article’s reported dataset can be found in Supplementary Materials.

## Supplementary Materials

The following supporting information can be downloaded at: https://www.mdpi.com/article/10.3390/s23031053/s1, Table S1: Dataset for ransomware and goodware features.
